# Development of an intervention to increase health service utilisation in ex-prisoners

**DOI:** 10.1186/2194-7899-2-4

**Published:** 2014-03-19

**Authors:** Stuart A Kinner, Kate van Dooren, Frances M Boyle, Marie Longo, Nicholas Lennox

**Affiliations:** 1 Melbourne School of Population and Global Health, The University of Melbourne, 207 Bouverie Street, Carlton, VIC 3010 Australia; 2 School of Medicine, The University of Queensland, 288 Herston Road, Herston, QLD 4006 Australia; 3 School of Public Health and Preventive Medicine, Monash University, 99 Commercial Road, Melbourne, VIC 3004 Australia; 4 Murdoch Children’s Research Institute, 50 Flemington Road, Parkville, VIC 3052 Australia; 5 Queensland Centre for Intellectual and Developmental Disability, School of Medicine, The University of Queensland, Raymond Terrace, South Brisbane, QLD 4010 Australia; 6 School of Population Health, The University of Queensland, Herston Road, Herston, QLD 4006 Australia; 7 Drug and Alcohol Services South Australia, 60 Marryatt Street, Port Adelaide, SA 5015 Australia

**Keywords:** Prisoner, Transition, Re-entry, Randomised controlled trial, Service brokerage, Case management, Intervention

## Abstract

The world prison population is growing at a rate well in excess of general population growth, with more than 10 million adults currently in custody around the world and around 30 million moving through prison systems each year. There is increasing recognition of the complex and chronic health needs of incarcerated populations, but evidence-based responses to these needs remain elusive. Most prisoners return to the community after a relatively short period of time in custody, however few transitional interventions for prisoners have been subjected to rigorous evaluation. This paper details the process of developing a service brokerage intervention for ex-prisoners in Queensland, Australia, and describes the resultant intervention. The intervention could be adapted for use in other settings and is amenable to methodologically rigorous evaluation. The collaborative design and development process involved extensive consultation with ex-prisoners and key government, community and consumer stakeholders. The intervention evolved considerably during the process of consultation, as we came to better understand the needs and priorities of our target population, and of the community organisations that served them. We consider genuine consultation with consumers, in a safe and supportive environment, to be an integral part of intervention research in this area. Given the poor outcomes experienced by many people after release from prison, evidence-based interventions developed in the way described here are urgently required.

**Electronic supplementary material:**

The online version of this article (doi:10.1186/2194-7899-2-4) contains supplementary material, which is available to authorized users.

## Background

The world prison population is growing at a rate well in excess of general population growth, with more than 10 million adults currently in custody around the world (Walmsley 2011) and around 30 million moving through prison systems each year (UNODC 2008). Australia accounts for only a small proportion of this total – just under 30,000 on any given day – although the rate of imprisonment in Australia has increased by 9% over the past decade (ABS 2012) and due to the rapid turnover of prisoners serving short sentences, around 55,000 adults move through Australia’s prisons each year (Martire and Larney 2012). One striking feature of Australia’s prison population is the over-representation of Indigenous people, who comprise around 2.5% of the Australian population but 27% of adult prisoners (ABS 2012).

There is increasing recognition of the complex and chronic health needs of incarcerated populations. Despite their relative youth, prisoners experience impaired general health on a range of measures (Butler et al. 2007), and the prevalence of blood-borne viruses, particularly HIV and hepatitis C, is typically orders of magnitude higher than in the community (Butler et al. 2011; Dolan et al. 2007; Macalino et al. 2004; Weinbaum et al. 2005). The prevalence of mental illness is similarly elevated, particularly for post-traumatic stress disorder, psychotic disorders and substance use disorders (Butler et al. 2006; Fazel and Danesh 2002; Heffernan et al. 2012). These complex and interconnected health problems are set against a backdrop of entrenched poverty and relative social disadvantage (Baldry et al. 2003; Social Exclusion Unit 2002; Travis and Petersilia 2001).

Although there is evidence that incarceration can both exacerbate mental illness (Douglas et al. 2009; WHO 2008) and precipitate risky drug use (Dolan et al. 2010; Dolan et al. 1996), for the most part prisoners bring their health problems with them from the community. For many, health improves while in custody, where food and accommodation are provided in a highly structured setting, illegal drugs are less readily available, and health services are provided at a level well in excess of that found in most communities (Feron et al. 2005; Marshall et al. 2001). However, almost all prisoners return to the community, with most of these ill-equipped to deal with their often complex health and psychosocial needs, and many returning to pre-incarceration patterns of behaviour and associated health outcomes within a relatively short period (Baldry et al. 2003; Hobbs et al. 2006; Kinner 2006; Visher and Courtney 2007). Two notable consequences of this predictable cycle are (a) high rates of recidivism (ABS 2012; SCRGSP 2011), and (b) a high incidence of preventable mortality, particularly in the first few weeks after release (Binswanger et al. 2007; Kinner et al. 2011; Seaman et al. 1998). Longitudinal studies of ex-prisoners have identified a high incidence of health risk behaviours (particularly risky drug use) and a low incidence of health seeking behaviours, including substantial under-utilisation of health services in the community, as potential targets for intervention (Kinner 2006; Kinner et al. 2012; Wang et al. 2008).

However, increasing health service utilisation in ex-prisoners is complex and presents numerous practical, social and systemic barriers (Kinner and Williams 2006). Practical barriers may include a lack of transport (particularly in regional and remote areas), insufficient identification documents to permit enrolment in some programs and services, and the sometimes prohibitive cost of treatment. Ex-prisoners may be unaware of the services available to them, particularly those whose unstable living circumstances preclude the development of community ties (Baldry et al. 2003). Even when services are available, most ex-prisoners have an imperative to address more immediate concerns (e.g., housing, food) before addressing ‘higher-level’ needs (Krieg 2006).

Ex-prisoners may also face psychosocial barriers to service access, with many suffering from a lack of motivation and perceiving that their situation is hopeless; a view sometimes reinforced by burnt-out frontline workers who (consistent with the paucity of empirical evidence) believe that ‘nothing works’ (Edelwich and Brodsky 1980). Many ex-prisoners are reluctant to access community services, feeling a sense of shame driven by the stigma of being an ‘ex-con’ and compounded by a reluctance to ask for help. For those who do seek support, funding limitations often restrict the capacity of service providers. A failure to accommodate the diversity that exists within the ex-prisoner population can result in an unhelpful homogeneity of services that ignores the unique needs of particular groups such as women and those from culturally and linguistically diverse backgrounds.

Finally, there are significant systemic barriers to service access for ex-prisoners. At least in the Australian context, there is often uncertainty about the date of release from custody, which complicates transitional planning and adds an extra element of stress to the release experience. Once released, ex-prisoners may be excluded from some services as a result of inflexible and unworkable eligibility criteria: key examples include the exclusion of those with active mental illness from substance treatment, and the wholesale exclusion of those with a history of violence from many treatment services. Furthermore, many ex-prisoners return to areas of relative social deprivation, where at least in the primary care setting they can expect shorter consultations, less health promotion and a greater emphasis on the prescription of (sometimes unaffordable) medications (Furler et al. 2002).

In recognition of these challenges, correctional systems in some countries have sought to develop and implement transitional programs to reduce health risk behaviour and increase access to services after release from prison (Borzycki 2005; Burrows et al. 2000). However, published evaluations of such programs are rare (Farrington & Welsh 2005; Petersilia 2004). One of the first to be rigorously evaluated was Project Greenlight, a quasi-randomised trial of an intervention delivered to a sample of prisoners in New York State in the eight weeks before their release from custody. One component of the intervention was service referral and although the primary aim of the intervention was to reduce recidivism, it also measured a range of health and psychosocial outcomes including substance use, family relationships and service access. Although the intervention increased service utilisation it did not improve health outcomes and *increased* recidivism, probably due to a combination of poor implementation, a mismatch between program intensity and target population, and failure to continue the intervention after release from custody (Wilson and Davis 2006). Two other US-based programs – Project START and the Serious and Violent Offender Re-entry Initiative (SVORI) – have produced more promising results (Lattimore and Visher 2009; Wolitski et al. 2006). Although quite different in structure and focus, both programs involved facilitating access to community services, flexibility in program implementation to tailor the program to individual strengths and needs, and recognition of health as important in its own right (rather than purely a corollary of reduced recidivism). Consistent with evidence of the crucial importance of providing support after release from custody (Kurlychek and Kempinen 2006), both programs were also distinguished by a focus on the provision of support *after release*.

Informed by this literature, and recognising that programs effective in the US context may not be appropriate in other settings, we sought to develop and evaluate an intervention to improve health outcomes for ex-prisoners in Queensland, Australia. Our aim was to develop a service brokerage intervention likely to have a positive effect on the health of ex-prisoners that could be adapted for use in other prison settings and that was amenable to methodologically rigorous evaluation. In this paper we describe the process of developing the intervention, describe the intervention itself and, where relevant, consider issues of evaluation design and implementation. The intervention is being evaluated in a single-blinded, randomised controlled trial, the design of which is described in detail elsewhere (Kinner et al. 2013). As one of the first transitional interventions for prisoners to be subjected to such rigorous evaluation, we hope that others might benefit from our experience.

## Methods

The aim of the Passports project was to develop a transitional intervention and evaluate its impact on post-release health service utilisation, physical and mental health, health-related quality of life and health risk behaviours among ex-prisoners, within the first six months of release; and the incidence and timing of recidivism within two years of release. The project emerged from a program of research that commenced in 2004 with a small longitudinal study of ex-prisoners in Queensland, Australia (Kinner 2006). This pilot study identified key health issues for ex-prisoners, tested measures of key constructs and most importantly, demonstrated the viability of strategies for recruiting and maintaining contact with ex-prisoners. Once funding for the Passports project was secured, project development proceeded over the course of a year, through four overlapping stages.

### Obtaining ethics approval and registering as a clinical trial

Funding for the Passports project was provided by the Australian National Health and Medical Research Council (NHMRC) under a special initiative focussed on ‘Preventive Healthcare and Strengthening Australia’s Social and Economic Fabric’. Given the over-representation of Indigenous people in Australia’s prisons (ABS 2012), the proposal was also reviewed and endorsed by the NHMRC’s Indigenous Health Research Panel. After institutional ethics approval was granted and funds were released, a project manager was appointed, a second approval was sought and obtained from the Queensland Corrective Services (QCS) Research Committee, and the trial was registered with the Australian New Zealand Clinical Trials Registry (ACTRN12608000232336).

### Refining study design

Study design was refined in an iterative fashion, based on a comprehensive review of the literature and informed by consultation with key government, community and consumer stakeholders. To this end, inclusive and diverse project advisory groups were established in the three sites (south-east Queensland; Townsville and Cairns over 1000 km to the north in Queensland) where recruitment was to take place. Although the majority of adult prisoners in Queensland are held in centres in the south-east corner of the state, we elected to also recruit participants from the north of the state (a) to ensure that the project had statewide relevance, and (b) to ensure adequate recruitment of Indigenous participants, who constitute the majority of prisoners in northern prisons. In each site, community stakeholders were identified through existing networks and using snowballing methods. Consumers were identified through community partners and other appropriate community contacts.

A critical part of this process was establishing whether it was feasible and ethical to implement a meaningful intervention within a randomised controlled trial (RCT) framework; key to gaining acceptance for the study was assuring stakeholders that the control arm would receive ‘usual care’ and that there was a *prima facie* case for equipoise. Stakeholders important to the study were diverse and included both government and non-government bodies providing services to prisoners and ex-prisoners (e.g., mental health, alcohol and other drug treatment, employment and housing, legal services), and non-government organisations serving advocacy functions. Reconciling the sometimes competing agendas of these diverse organisations was challenging but a shared commitment to improving outcomes for ex-prisoners provided some common ground.

The process of educating stakeholders about the research design was an essential part of the development process. As proposed methodological refinements were generated, these were presented to stakeholders and consumers (former prisoners) who commented on their viability and acceptability within the correctional and post-release environments. Through this iterative process the final study design reflected both best practice from a methodological point of view and a workable, ethically sound model from a stakeholder and consumer point of view.

### Development of intervention methods and materials

Stakeholders endorsed the research team’s commitment to focussing on post-release support, and to ensuring that the intervention complemented an existing pre-release program delivered by Queensland Corrective Services (QCS). The existing ‘Transitions’ program consisted of a series of ‘modules’ delivered in prison, primarily by community service providers; a small subset of prisoners also received some post-release support (QCS 2009). Stakeholders observed that (a) at that time the Transitions program was available for only a subset of prisoners and had never been evaluated, and (b) consistent with evidence from US studies, an optimal approach would involve engaging with participants before release from custody, and continuing this engagement post-release.

The Passports intervention had its origins in an intervention developed through a similar collaborative process, but intended to improve communication between patients with intellectual disability and their general practitioners (Lennox et al. 2004a). We chose this intervention as a starting point because it was designed for a vulnerable population who tend to under-utilise health care, and it was developed through an iterative, collaborative process that actively involved consumers.

Based on a review of the literature and our knowledge of the population and context, we developed a first draft of the proposed intervention materials and protocols. Using an iterative approach, this and subsequent drafts of the intervention protocols and materials were presented to stakeholders during semi-structured meetings and informal consultations; feedback was carefully documented and incorporated into subsequent drafts. To ensure meaningful input from consumers, in addition to consumer representatives on our stakeholder groups, separate focus groups comprised exclusively of ex-prisoners were convened in the three study sites. These focus groups were facilitated by project staff with the support of a local non-government agency. Feedback from these consumer focus groups resulted in substantial changes to the intervention materials, with the end result striking a balance between what was considered theoretically important to improving health outcomes for ex-prisoners, and what was considered acceptable and relevant (i.e., face validity) by consumers.

In addition to consulting with stakeholder and consumer groups, the intervention was informed by qualitative interviews with 24 ex-prisoners from diverse backgrounds, who had spent at least two years in the community prior to interview. Participants in these interviews were identified through relevant community agencies and provided written, informed consent. Interviews were audio-recorded and transcribed, and analysed thematically. As well as informing the development of the intervention (van Dooren et al. 2012), the findings from this qualitative work were incorporated into the intervention itself, in the form of brief stories of change written by the participants (see Table 1).

**Table 1:** Elements of the intervention

Although essentially a post-release intervention, the Passports intervention begins when the participant receives their intervention materials, which are placed with their other personal belongings to be collected at the point of release. Although it may have been beneficial to provide the Passport *prior* to the point of release, we suspected that this would introduce an unacceptable degree of contamination. Following extensive consultation with stakeholders and consumers, the content and format of the intervention were finalised, and comprised the following:	
1. *Kitbag*	A practical issue identified by consumers and some community stakeholders was that many prisoners leaving custody were provided with only a plastic garbage bag for their personal belongings, which was perceived as both impractical and stigmatising. For less than AU$10 per participant we purchased mid-sized, unmarked backpacks in which participants could place their belongings on release. Our intervention materials were placed in the bag, reducing the risk of them being discarded or lost. The kitbag had high face validity and was very well received by participants.
2. *Cover letter*	The kitbag contained a cover letter addressed to the participant and written in plain language, explaining the contents of the kit and the nature and extent of support available from the Passports team. The cover letter also identified dates for follow-up contact in the community and encouraged the participant to call the research team on a freecall 1800 number to keep their contact details up to date. Forms and reply paid envelopes were also provided for this purpose.
3. *The Passport*	The core of the intervention was the ‘Passport’ – a personalised booklet consisting of (a) a step-by-step checklist for the participant to work through post-release (e.g., getting identity documents and a bank account, finding housing and a GP), generated and refined in consultation with consumers; (b) a visually-based, user-friendly summary of the participant’s health status (e.g., hepatitis C infection, medications), including a removable health summary that could be provided to a health professional; and (c) a personalised list of services (e.g., doctor, sexual health, counselling, housing, employment, needle exchange) in the community, tailored to age, gender, Indigenous status, alcohol and other drug use history and other personal and life circumstances. The list of services was extensively tailored to the individual, including to their location anywhere in the state of Queensland (an area of 1.7 million square kilometres) and in some cases interstate. The booklets were passport-sized so that they fit easily in a pocket and bound in a vinyl cover so that they were robust. Although the participant’s first name appeared in a number of places, the Passport did not contain sufficient information to permit identification of the individual, should it be misplaced or stolen. Participants were routinely reminded that a replacement Passport could be sent to them at any time, at no cost, and a number of participants requested and received replacement Passports during the trial.
4. *Common Threads*	Development of the intervention was informed by qualitative interviews with 24 ex-prisoners. In collaboration with the research team, these 24 individuals reduced transcripts of their interviews to brief, de-identified stories of change, presented in a visually appealing way and in plain language, and highlighting key challenges and how they were overcome. These stories were bound together in a book published for the project and entitled *Common Threads*.
5. *Telephone support*	Following release, intervention participants received telephone calls from trained staff once a week for the first four weeks. Wherever possible, each call was made by the same staff member, to facilitate the development of rapport and trust. Although these calls were primarily for the purposes of providing support, and incorporated some key principles from motivational interviewing (Miller and Rollnick 2002), during each phone call data were collected across the six domains listed above. Intervention participants also had access to a freecall 1800 number they could call to seek advice, support and referral. All incoming calls to this number were logged and information about the nature and outcome of the call was recorded.
In addition to usual care, the control group received a brief letter (a) thanking them for participating, (b) providing a very brief summary of their health, identifying whether their assessment results were below average, average or above average in each domain, (c) reminding them that they would be contacted again one, three and six months post-release (and specifying dates for this), and (d) providing a phone number to contact the researchers if they wished to update their contact details.	

### Implementation

Poor implementation can dilute the impact of a well-designed intervention and compromise the validity of a well-planned evaluation. Particularly among vulnerable populations such as ex-prisoners, problems with implementation may even result in unintended, adverse outcomes (Wilson 2007; Wilson and Davis 2006). The project team was committed to maintaining the quality of the intervention and the integrity of its evaluation, recognising that robust evidence for change was contingent on both. Key to our success in this endeavour was the collaborative relationship between the project team and QCS, who served as gatekeepers, advisors and facilitators throughout the project. Protocols were designed to maintain the integrity of the intervention and evaluation while minimising impact on QCS staff and prisoners. Although the former took precedence, our efforts regarding the latter earned us the respect and gratitude of QCS staff, so that the project came to be seen as a collaborative venture rather than an imposition by researchers on the correctional system. Nonetheless, independence from QCS was equally valuable, allowing our interviewers to distinguish themselves from correctional staff and affording us the freedom to make our findings publicly available, regardless of the outcome.

A second key issue with respect to implementation related to recruitment and training of project staff. Training was comprehensive and multi-faceted and included issues relating to the health of justice-involved populations, Indigenous concepts of health and well-being, mental health, intellectual disability and principles of motivational interviewing (Miller and Rollnick 2002), as well as protocols around duty of care and ethical requirements. Ongoing adherence to study protocols was achieved through regular meetings and re-training as required, including the development of systems for monitoring progress in recruitment, intervention and evaluation. Staff were also provided with ongoing support and debriefing where appropriate.

## Results

A comprehensive review of the literature revealed a large number of papers discussing transitional and post-release issues, a smaller number presenting cross-sectional descriptions of samples of ex-prisoners, fewer still reporting the results of longitudinal studies of ex-prisoners, and only a handful describing or reporting any sort of evaluation of a transitional intervention. There was therefore little in the literature to guide development of the Passports intervention, however we benefited from work documenting service brokerage interventions for other populations including people with an intellectual disability (Lennox et al. 2004a; Lennox et al. 2004b) and people recently diagnosed with HIV (Gardner et al. 2005).

**Figure 1:**
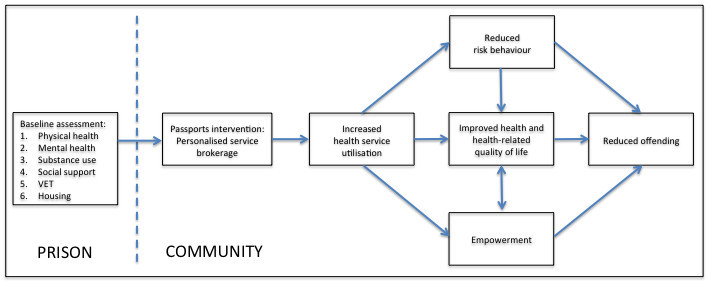
**Conceptual framework underpinning the Passports intervention.**

The conceptual framework underpinning the Passports intervention is presented in Figure 1. Although the intervention is flexible and tailored to the individual, it includes a focus on functioning in six domains that mirror the domains targeted by the existing Transitions intervention provided by QCS: physical health, mental health, substance use, social support and community engagement, vocational education and training (VET) and housing. In each of these domains, the individual’s strengths and needs are identified through a comprehensive pre-release assessment that informs direct health promotion activities and efforts to facilitate utilisation of appropriate services in the community, post-release. The proximate aim of the intervention is to increase utilisation of appropriate community services, which may in turn reduce health risk behaviour, promote empowerment and thereby achieve sustainable improvements in health. Reduced offending may be a desirable corollary of these outcomes.

The intervention was embedded in a single-blinded, multi-site RCT design. The evaluation involved baseline interviews within six weeks of expected release from prison and before randomisation; and follow-up telephone interviews one, three and six months after release from prison. Two-year recidivism data were obtained from QCS. In addition, participants consented to the research team accessing their prison health records and Medicare records to examine patterns of health care utilisation in custody and in the two years following release from prison. The evaluation design is described in more detail elsewhere (Kinner et al. 2013).

## Discussion

In this paper we have described the process of developing a service brokerage intervention for adult prisoners returning to the community in Queensland, Australia. The intervention was informed by the literature, underpinned by a collaborative relationship with corrective services and refined through a year-long process of consultation with community stakeholders and consumers. The end result was a unique, low-cost intervention focussed on facilitating access to existing community services. Crucially, the intervention was designed and implemented in a way that permitted rigorous evaluation through an RCT design.

Few transitional interventions for prisoners have been subjected to rigorous evaluation (Petersilia 2004) and not all have produced favourable results (Wilson and Davis 2006; Wohl et al. 2011). This dearth of evidence was a key reason for undertaking the Passports study but provided limited guidance in developing the intervention. Informed by the extant literature, we sought to develop an intervention that could be tailored to the individual, and that focussed on providing post-release support through facilitating access to existing community services. Although the final intervention remained true to these principles it evolved considerably during the process of stakeholder and consumer consultation, as we came to better understand the needs and priorities of our target population and of the community organisations that served them. The consultation process was particularly valuable in identifying important practical considerations, for example the need for the Passport to fit in a back pocket, the advantages of delivering the intervention materials in an unmarked backpack, and strategies for minimising literacy concerns. We consider genuine consultation with consumers in a safe and supportive environment to be an integral part of intervention research in this area (Canadian HIV/AIDS Legal Network 2006).

Understanding whether an intervention is likely to be effective in everyday practice also requires a detailed understanding of the contexts in which the intervention will take place and the range of potential barriers and enablers at individual, organisational and wider systems and policy levels (Craig et al. 2008; MRC 2008). The Passports study benefited from a long history of collaboration between the research team and QCS, which ensured that our intervention complemented rather than duplicated existing programs. Equally, our independence from QCS allowed us to operate outside of existing policy frameworks, and was a prerequisite for implementing the intervention in an RCT design. Accessing incarcerated populations for research purposes requires the support of correctional authorities, who operate in a challenging and highly politicised environment. Striking a balance between collaboration and independence in this area can be challenging, however in our experience mutual respect was the basis for a productive collaboration.

Implementation fidelity is critical to the success of any intervention. Our approach to implementation of the intervention provided for routine monitoring of program fidelity through careful documentation of milestones and weekly review of implementation challenges and successes. This process was made easier by the fact that our intervention was delivered primarily in the community rather than in prison.

### Future directions

The Passports intervention was designed to complement an existing transitional intervention in Queensland and as such would require adaptation to be appropriate for use in other settings. Just as stakeholder and consumer input was essential in developing the intervention, we would encourage those aiming to adapt this intervention to other settings to work collaboratively with community stakeholders and consumers, as well as the relevant correctional authority, in their jurisdiction.

An advantage of service brokerage interventions in general, and the Passports intervention in particular, is their relatively low cost. Rather than developing a new service our intervention consisted of identifying the participant’s needs and assisting them to identify existing community services that could help them to meet those needs. The most resource-intensive component of the intervention was developing personalised lists of services for each participant, which necessitated both considerable staff time and costs in purchasing access to service databases. In addition to examining the impact of the intervention on health service utilisation, health and offending outcomes, we aim to examine the cost-effectiveness of the Passports intervention, taking into account development and implementation costs, and cost savings associated with improved health-related quality of life, and avoided health problems and incarceration costs. In a context of fiscal conservatism and little appetite for increased expenditure on an already marginalised and stigmatised population, we believe that evidence of cost-effectiveness will strengthen any argument for increased support for people transitioning from prison to the community. Given the poor outcomes experienced by many people after release from prison, evidence-based, cost-effective interventions to improve health outcomes for ex-prisoners are clearly needed.

## Authors’ original submitted files for images

Below are the links to the authors’ original submitted files for images. Authors’ original file for figure 1

## References

[CR1] ABS (2012). Prisoners in Australia 2012.

[CR2] Baldry E, McConnell D, Maplestone P, Peeters M (2003). Ex-prisoners and accommodation: What bearing do different forms of housing have on social reintegration?.

[CR3] Binswanger IA, Stern MF, Deyo RA, Heagerty PJ, Cheadle A, Elmore JG, Koepsell TD (2007). Release from Prison — A High Risk of Death for Former Inmates. New England Journal of Medicine.

[CR4] Borzycki M (2005). Interventions for prisoners returning to the community.

[CR5] Burrows J, Clarke A, Davison T, Tarling R, Webb S (2000). Research Findings. The nature and effectiveness of drugs throughcare for released prisoners.

[CR6] Butler T, Andrews G, Allnutt S, Sakashita C, Smith NE, Basson J (2006). Mental disorders in Australian prisoners: a comparison with a community sample. Australian and New Zealand Journal of Psychiatry.

[CR7] Butler T, Kariminia A, Levy M, Murphy M (2007). The self-reported health status of prisoners in New South Wales. Australian and New Zealand Journal of Public Health.

[CR8] Butler T, Lim D, Callander D (2011). National prison entrants’ bloodborne virus & risk behaviour survey 2004, 2007 and 2010.

[CR9] Canadian HIV/AIDS Legal Network (2006). “Nothing about us without us”: Greater, meaningful involvement of people who use illegal drugs: A public health, ethical, and human rights imperative.

[CR10] Craig P, Dieppe P, Macintyre S, Michie S, Nazareth I, Petticrew M (2008). Developing and evaluating complex interventions: the new Medical Research Council guidance. British Medical Journal.

[CR11] Dolan K, Kite B, Black E, Aceijas C, Stimson GV, Reference Group on HIV AIDS Prevention and Care among Injecting Drug Users in Developing and Transitional Countries (2007). HIV in prison in low-income and middle-income countries. The Lancet Infectious Diseases.

[CR12] Dolan K, Teutsch S, Scheuer N, Levy M, Rawlinson W, Kaldor J, Lloyd A, Haber P (2010). Incidence and risk for acute hepatitis C infection during imprisonment in Australia. European Journal of Epidemiology.

[CR13] Dolan K, Wodak A, Hall W, Gaughwin M, Rae F (1996). HIV risk behaviour of IDUs before, during and after imprisonment in New South Wales. Addiction Research.

[CR14] Douglas N, Plugge E, Fitzpatrick R (2009). The impact of imprisonment on health: what do women prisoners say?. Journal of Epidemiology and Community Health.

[CR15] Edelwich J, Brodsky A (1980). Burn-out: Stages of disillusionment in the helping professions.

[CR16] Farrington DP, Welsh BC (2005). Randomized experiments in criminology: What have we learned in the last two decades?. Journal of Experimental Criminology.

[CR17] Fazel S, Danesh J (2002). Serious mental disorder in 23 000 prisoners: a systematic review of 62 surveys. The Lancet.

[CR18] Feron JM, Paulus D, Tonglet R, Lorant V, Pestiaux D (2005). Substantial use of primary health care by prisoners: epidemiological description and possible explanations. Journal of Epidemiology and Community Health.

[CR19] Furler JS, Harris E, Chondros P, Powell Davies PG, Harris MF, Young DY (2002). The inverse care law revisited: impact of disadvantaged location on accessing longer GP consultation times. The Medical Journal of Australia.

[CR20] Gardner LI, Metsch LR, Anderson-Mahoney P, Loughlin AM, Rio C d, Strathdee S, Sansom SL, Siegal HA, Greenberg AE, Holmberg SD, Antiretroviral Treatment and Access Study Study Group (2005). Efficacy of a brief case management intervention to link recently diagnosed HIV-infected persons to care. AIDS.

[CR21] Heffernan E, Andersen K, McGrath J, Dev A, Kinner SA (2012). Mental illness is highly prevalent among Aboriginal and Torres Strait Islander people in custody. The Medical Journal of Australia.

[CR22] Hobbs M, Krazlan K, Ridout S, Mai Q, Knuiman M, Chapman R (2006). Mortality and morbidity in prisoners after release from prison in Western Australia 1995–2003.

[CR23] Kinner SA (2006). Continuity of health impairment and substance misuse among adult prisoners in Queensland, Australia. International Journal of Prisoner Health.

[CR24] Kinner SA, Lennox N, Williams GW, Carroll M, Quinn B, Boyle F, Alati R (2013). Randomised controlled trial of a service brokerage intervention for ex-prisoners in Australia. Contemporary Clinical Trials.

[CR25] Kinner SA, Milloy M-J, Wood E, Qi J, Zhang R, Kerr T (2012). Incidence and risk factors for non-fatal overdose among a cohort of recently incarcerated illicit drug users. Addictive Behaviors.

[CR26] Kinner SA, Preen D, Kariminia A, Butler T, Andrews J, Stoové M, Law M (2011). Counting the cost: Estimating of the number of deaths among recently released prisoners in Australia. Medical Journal of Australia.

[CR27] Kinner SA, Williams M (2006). Post-release experience of prisoners in Queensland: implications for community and policy.

[CR28] Krieg AS (2006). Aboriginal incarceration: health and social impacts. [Viewpoint]. Medical Journal of Australia.

[CR29] Kurlychek M, Kempinen C (2006). Beyond boot camp: The impact of aftercare on offender reentry. Criminology and Public Policy.

[CR30] Lattimore PK, Visher CA (2009). The multi-site evaluation of SVORI: Summary and synthesis: National Institute of Justice.

[CR31] Lennox N, Rey-Conde T, Bain C, Purdie D, Bush R (2004). The evidence for better health from health assessments: A large clustered randomised controlled trial. [published abstract]. Journal of Intellectual Disability Research.

[CR32] Lennox N, Taylor M, Rey-Conde T, Bain C, Boyle FM, Purdie DM (2004). Ask for it: Development of a health advocacy intervention for adults with intellectual disability and their general practitioners. Health Promotion International.

[CR33] Macalino GE, Hou JC, Kumar MS, Taylor LE, Sumantera IG, Rich JD (2004). Hepatitis C infection and incarcerated populations. International Journal of Drug Policy.

[CR34] Marshall T, Simpson S, Stevens A (2001). Use of health services by prison inmates: comparisons with the community. Journal of Epidemiology and Community Health.

[CR35] Martire KA, Larney S (2012). Increasing numbers of inmate separations from Australian prisons. The Medical Journal of Australia.

[CR36] Miller WR, Rollnick S (2002). Motivational interviewing: Preparing people for change.

[CR37] MRC (2008). Developing and evaluating complex interventions: new guidance.

[CR38] Petersilia J (2004). What Works in Prisoner Reentry - Reviewing and Questioning the Evidence. Federal Probation.

[CR39] QCS (2009). Transitions: Release preparation program (Fact Sheet).

[CR40] SCRGSP (2011). Report on Government Services 2011.

[CR41] Seaman SR, Brettle RP, Gore SM (1998). Mortality from overdose among injecting drug users recently released from prison: database linkage study. British Medical Journal.

[CR42] Social Exclusion Unit (2002). Reducing re-offending by ex-prisoners (pp. 218).

[CR43] Travis J, Petersilia J (2001). Reentry Reconsidered: A New Look at an Old Question. Crime & Delinquency.

[CR44] UNODC (2008). Prevention of spread of HIV amongst vulnerable groups in South Asia.

[CR45] van Dooren K, Claudio F, Kinner SA, Williams M (2012). Beyond reintegration: a framework for understanding ex-prisoner health. International Journal of Prisoner Health.

[CR46] Visher CA, Courtney SME (2007). One year out: Experiences of prisoners returning to Cleveland Returning home policy brief.

[CR47] Walmsley R (2011). World prison population list.

[CR48] Wang EA, White MC, Jamison R, Goldenson J, Estes M, Tulsky JP (2008). Discharge planning and continuity of health care: findings from the San Francisco County Jail. American Journal of Public Health.

[CR49] Weinbaum CM, Sabin KM, Santibanez SS (2005). Hepatitis B, hepatitis C, and HIV in correctional populations: a review of epidemiology and prevention. AIDS.

[CR50] WHO (2008). Trencin statement on prisons and mental health.

[CR51] Wilson JA (2007). Habilitation or harm: Project Greenlight and the potential consequences of correctional programming. NIJ Journal.

[CR52] Wilson JA, Davis RC (2006). Good intentions meet hard realities: An evaluation of the Project Greenlight reentry program. Criminology & Public Policy.

[CR53] Wohl DA, Scheyett A, Golin CE, White B, Matuszewski J, Bowling M, Smith P, Duffin F, Rosen D, Kaplan A, Earp J (2011). Intensive case management before and after prison release is no more effective than comprehensive pre-release discharge planning in linking HIV-infected prisoners to care: a randomized trial. AIDS and Behavior.

[CR54] Wolitski RJ, Project START Writing Group for the Project START Study Group (2006). Relative Efficacy of a Multisession Sexual Risk– Reduction Intervention for Young Men Released from Prisons in 4 US States. American Journal of Public Health.

