# Supplementary figures and images for: Development of an intervention to increase health service utilisation in ex-prisoners
Source: Health Justice. 2014 Mar 19;2:4. doi: 10.1186/2194-7899-2-4 (PMC5151804; doi:10.1186/2194-7899-2-4)

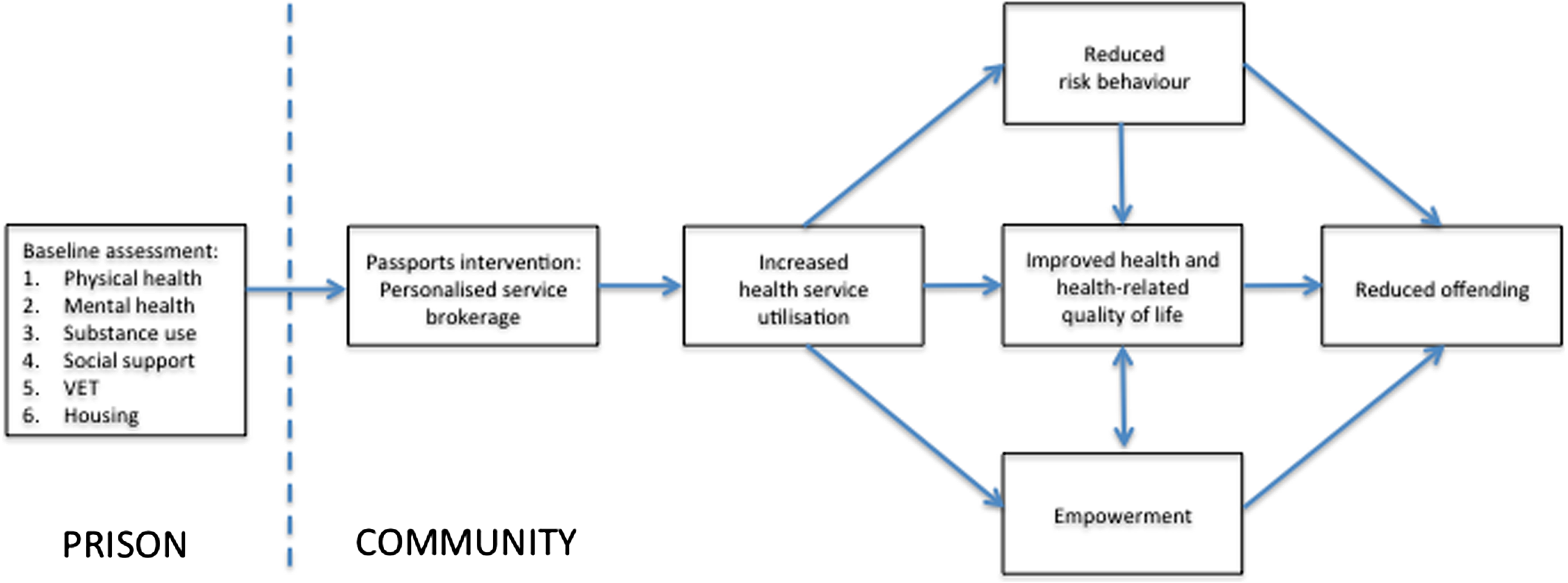

Supplement: Supplementary file 1 — Authors’ original file for figure 1 [file 40352_2013_4_MOESM1_ESM.tiff]
